# Reversing the Norm: Successful Cholecystectomy in a Patient With Situs Inversus

**DOI:** 10.7759/cureus.59957

**Published:** 2024-05-09

**Authors:** Bahaa Nassr, Hasan Nassr, Abdullah Allouzi, Abubakar Abdalla, Talal Shaheen, Ammar Alkhatabi, Osama Alkhatabi

**Affiliations:** 1 General Surgery and Surgical Oncology, Al Salam Health Medical Hospital, Riyadh, SAU; 2 College of Medicine, Sulaiman Alrajhi University, Al Bukairiyah, SAU; 3 General Surgery, Al Salam Health Medical Hospital, Riyadh, SAU

**Keywords:** surgery, hepatobiliary, chronic calculous cholecystitis, laparoscopic cholecystectomy, situs inversus

## Abstract

Situs inversus totalis (SIT), affecting 1 in 6,000 to 10,000 individuals, involves a complete reversal of chest and abdominal organs. About one-third of SIT cases coincide with primary ciliary dyskinesia, leading to diverse symptoms. Surgical challenges arise in procedures like liver transplantation and biliary interventions due to organ abnormalities. This case study explores cholecystitis in a patient with SIT, offering insights crucial for navigating complexities in treating this congenital anomaly. A 34-year-old Arab female, who was a known SIT case, came to the hospital complaining of abdominal pain in the left upper quadrant. After conducting a chest X-ray and an abdominal ultrasound, the patient was diagnosed with cholecystitis. She then underwent a planned cholecystectomy to remove her gallbladder. SIT presents challenges when it comes to procedures such as laparoscopic cholecystectomy (LC). Nevertheless, the proficiency of skilled surgeons, meticulous preoperative planning, and strict adherence to surgical principles render the execution of LC on patients with SIT both achievable and secure. The successful completion of over 120 cases serves as evidence of the adaptability and precision that can be achieved through surgery for individuals with SIT.

## Introduction

Situs solitus is the medical term used to describe the abdominal and thoracic organs when they are stationed in their normal anatomical positions. However, the complete reversal of organ position (mirror-image transposition) within the thoracic and abdominal cavities is called situs inversus totalis (SIT), which is an exceedingly rare congenital anomaly [1]. When it comes to the prevalence, SIT ranges between 1:6,000 and 1:10,000, making it a true medical curiosity, and it has been reported that approximately one-third of those patients have primary ciliary dyskinesia, which is an autosomal recessive genetic ciliopathy [1,2].

SIT manifests differently depending on the individual, ranging from those who are asymptomatic and remain unaware of their condition until an incidental discovery during a medical examination to those with congenital cardiac defects or respiratory complications associated with the condition [1,2]. Such a condition is challenging for surgeons since there are frequent malformations in the transposed organs along with vascular and anatomical variations. At the top of the list of challenging surgeries are liver transplantation and resection, followed by portal vein embolization and percutaneous biliary procedures [1,3].

In this paper, we will describe a case of chronic calculous cholecystitis in a patient with SIT who underwent laparoscopic cholecystectomy (LC). We will discuss the pathological, radiological, and surgical findings, as well as the management plan for the patient.

## Case presentation

A 34-year-old Arab female patient, previously diagnosed with SIT, presented to the outpatient general surgery clinic with moderate left upper quadrant pain. The pain started three weeks before her presentation to the clinic. The pain, colicky in character, exacerbated every two to three hours and radiated to the back. The patient stated that on the subjective pain score, she evaluated the pain as 7 out of 10. The pain was not associated with fever, jaundice, anorexia, nausea, or vomiting. Moreover, no specific decubitus or movement diminishes or aggravates the pain. Systemic review of the patient’s complaints was unremarkable. The patient presented late to the clinic due to the over-the-counter usage of paracetamol (1,000 mg every six hours) since the abdominal pain started. Fifteen years ago, she was incidentally diagnosed with SIT when she experienced dyspnea, and her X-ray revealed this condition. There was no family history of SIT or other chronic or serious diseases.

On general physical examination, the patient’s vitals were all within normal ranges, establishing hemodynamic stability as follows: blood pressure 115/70 mmHg, respiratory rate 17 breaths/minute, pulse rate 87 beats/minute, and temperature 37 °C. On abdominal examination, the abdomen was soft and lax with no tenderness or guarding noted. Normal bowel sounds were heard upon auscultation.

**Figure 1:**
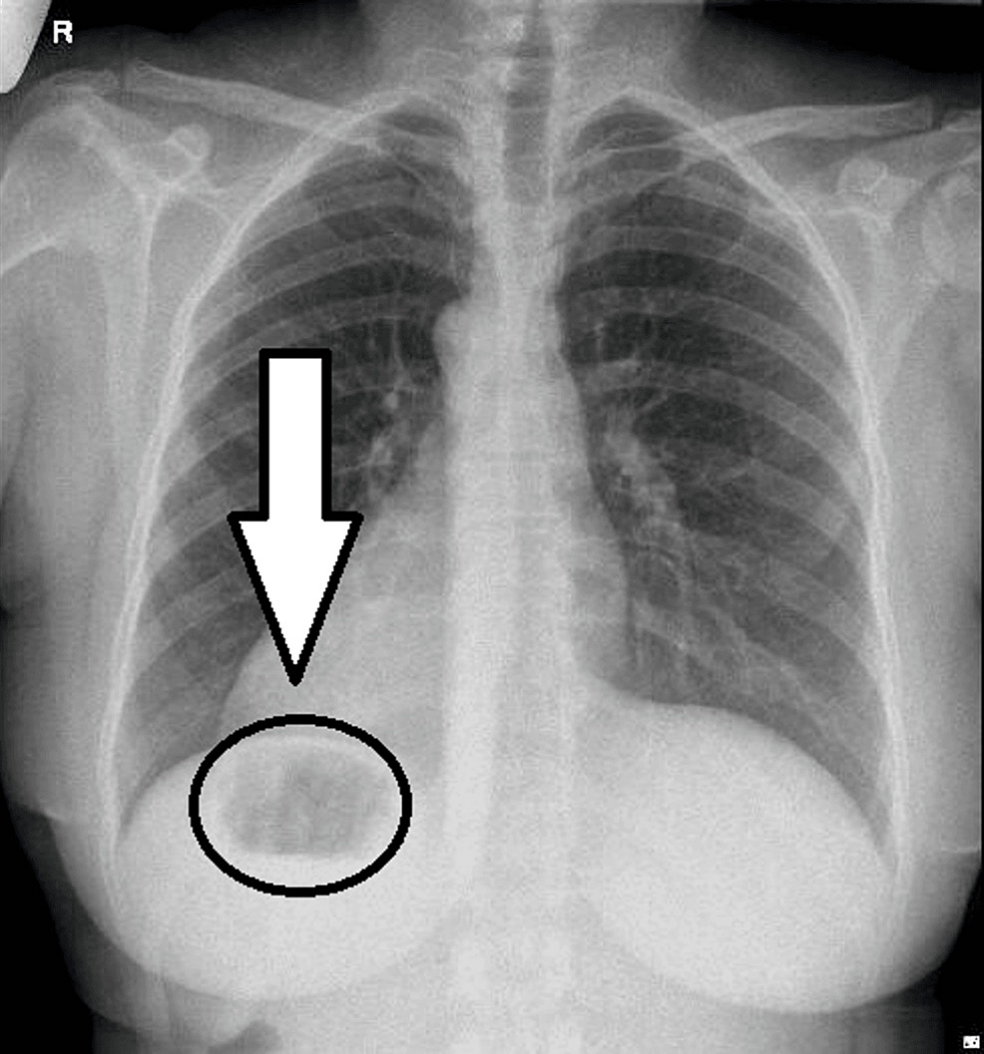
Posterior-anterior chest X-ray. The right-sided cardiac apex (shown by the arrow). Gastric gas under the right diaphragm (black circle).

**Figure 2:**
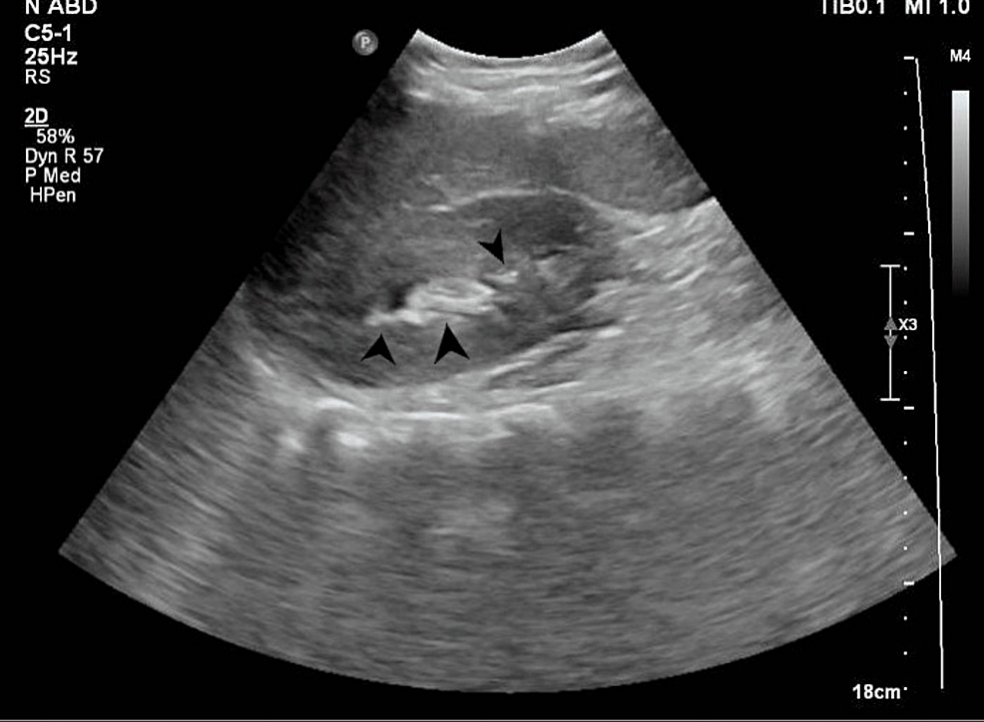
Abdominal ultrasound. Abdominal ultrasound showing a gallbladder with multiple stones (black arrowheads).

A posterior-anterior chest X-ray was done to confirm the diagnosis and assess the position of the heart and lungs (Figure 1). The radiograph showed a right-sided cardiac apex with a prominent gastric gas under the right hemidiaphragm. There were exaggerated bronchovascular markings, with an average cardiothoracic ratio. An abdominal ultrasound was ordered as well to assess the biliary organs. The ultrasound showed a left-sided gallbladder that was contracted over a large number of tiny echogenic stones (Figure 2). The common bile duct caliber was average, with no stones, masses, or constrictions observed. Additionally, there were no intra- or extra-hepatic biliary radical dilatations.

Preoperatively, a complete blood count (CBC), coagulation profile, total and direct bilirubin, blood type, and crossmatch were ordered. The CBC results were as follows: white blood cell count (WBC) 9.32 × 10^3^/µL (normal), red blood cell count (RBC) 4.42 × 10^12^/L (normal), hemoglobin 12.2 g/dL (normal), hematocrit 35.4% (low), platelet count 232 × 10^3^/µL (normal), and mean platelet volume (MPV) 11.7 fL (high). The coagulation profile, direct bilirubin, and total bilirubin results were all within normal limits.

An elective LC was done. Under general anesthesia and aseptic technique, the skin was draped. A supraumbilical, epigastric, left midclavicular, and left lateral anterior axillary ports were inserted, and the abdomen was insufflated by CO_2_ gas at a pressure of 15 mmHg. Upon exploration of the whole abdominal cavity, the liver is seen on the left side with an enlarged gallbladder (Video 1). The Calot’s triangle was successfully identified. The gallbladder was dissected from the surrounding omentum. Then, the cystic duct and artery were clipped and cut. Next, the gallbladder was dissected from the gallbladder bed from the liver by hook diathermy. Cholecystectomy was performed, and the gallbladder was extracted through the epigastric port. Finally, the gas was disinflated, and the ports were removed under supervision followed by closure of the port sites by stitches and dressing.

**Video 1:** Laparoscopic view. The laparoscopic view clearly shows the liver on the left side and the stomach on the right.

Pathological examination of the cholecystectomy revealed a gallbladder with multiple pieces measuring 6 cm x 1.5 cm x 0.5 cm. The mucosal surface was tan and velvety. The gallbladder contained multiple yellow irregular stones, ranging from 0.2 to 0.3 cm in greatest dimension. The wall of the gallbladder measured 0.2 cm in thickness. Microscopic examination of the sections revealed focal mucosal erosion, infiltrated by lymphocytes and plasma cells, confirming the diagnosis of chronic calculous cholecystitis.

The patient was discharged with the following medications: lornoxicam 8 gm (twice daily for one week), paracetamol 500 mg (three times daily for one week), and cefuroxime 500 mg (twice daily for one week). The patient had no postsurgical complications. Upon follow-up one week post-surgery, the patient reported no complaints.

## Discussion

SIT is an exceptional autosomal recessive disorder in which the whole body organs are reversed to the other side [1]. The entire mirror-image reversal of the abdominal and thoracic organs is the hallmark of SIT. Patients with SIT have their gallbladder situated on the left side of the abdomen under the ribs and their liver. Surgical procedures, especially those involving the biliary system such as cholecystectomy, are made more difficult by this anatomical diversity. It can be linked to several congenital abnormalities, including Yoshikawa's syndrome, which is defined by the coexistence of SIT, bilateral renal dysplasia, pancreatic fibrosis, and meconium ileus, and Kartagener's syndrome, which consists of the trio of SIT, sinusitis, and bronchiectasis [4].

It’s challenging to diagnose gallstones in patients with SIT due to the unusual presentation of the disease with the transposition of the body organs, causing a delay in the management and diagnosis, particularly in cases in which patients are not aware of having SIT [4]. The gold standard of care for treating symptomatic gallstones and disorders linked to the gallbladder is LC. The concepts of LC are essentially the same, even though the inverted anatomy may seem intimidating. Surgeons must, however, adjust to the changed anatomy, which makes this a fascinating and difficult process [5].

A crucial factor to take into account while treating LC in SIT is the requirement for meticulous preoperative planning and imaging. Planning the placement of the trocar and determining the anatomical variances need the use of CT scans and ultrasound exams. To correctly navigate and handle the instruments during the process, the surgeon must mentally replicate their understanding of the anatomy [5]. 

In the era before laparoscopic procedures became popular, open cholecystectomy was considered the approach, for treating gallstones [4,6]. There have been around 40 reported cases of open cholecystectomy in patients with SIT. However, the introduction of such an approach has greatly impacted surgical practices. Following the LC performed by Mouret in 1987, it quickly became the method for treating cholelithiasis [7]. In 1991, Campos and Sipes successfully performed the first cholecystectomy on a patient with SIT, and since then, more than 120 similar cases have been documented [8]. Remarkably, none of these cases experienced complications or required conversion to open cholecystectomy despite the challenges associated with performing laparoscopy on these patients [5-7]. The success can be attributed to surgeons conducting these surgeries and a documented track record of positive outcomes. Therefore, LC is considered a procedure that is not contraindicated in patients with SIT. Nonetheless, it presents difficulties due to the mirror-image anatomy that requires dissection of the biliary tree to minimize any unintended damage [6].

## Conclusions

In conclusion, when performed by experienced surgeons with a complete understanding of the reversed anatomy, LC in SIT is a realistic and safe technique. For effective outcomes, preoperative imaging, meticulous planning, and adherence to recognized surgical principles are vital. While the method may provide unique obstacles, it allows surgeons to display adaptability and precision in the field of minimally invasive surgery.

## References

[1] Eitler K, Bibok A, Telkes G (2022). Situs inversus totalis: a clinical review. Int J Gen Med.

[2] Dastouri D, McSweeney W, Sivananthan S (2022). Situs inversus: an interesting case of spontaneous splenic rupture. J Surg Case Rep.

[3] Zhang C, Zhang B, Huang H (2022). Situs inversus totalis with local metastasis of gallbladder carcinoma and variation of the common hepatic artery. BMC Gastroenterol.

[4] AlKhlaiwy O, AlMuhsin AM, Zakarneh E, Taha MY (2019). Laparoscopic cholecystectomy in situs inversus totalis: case report with review of techniques. Int J Surg Case Rep.

[5] Enciu O, Toma EA, Tulin A, Georgescu DE, Miron A (2022). Look beyond the mirror: laparoscopic cholecystectomy in situs inversus totalis- a systematic review and meta-analysis (and report of new technique). Diagnostics (Basel).

[6] Suleimanov V, Al Asker H, Al Hawaj K, Alhashim IW, Al Rebh FN (2022). Laparoscopic cholecystectomy in a morbidly obese patient with situs inversus totalis: a case report. Cureus.

[7] Borgaonkar VD, Deshpande SS, Kulkarni VV (2011). Laparoscopic cholecystectomy and appendicectomy in situs inversus totalis: a case report and review of literature. J Minim Access Surg.

[8] Campos L, Sipes E (1991). Laparoscopic cholecystectomy in a 39-year-old female with situs inversus. J Laparoendosc Surg.

